# Circulating miRNAs as biomarkers for the diagnosis in patients with melanoma: systematic review and meta-analysis

**DOI:** 10.3389/fgene.2024.1339357

**Published:** 2024-02-14

**Authors:** Nicholas Jones, Taichiro Nonaka

**Affiliations:** ^1^ School of Medicine, Louisiana State University Health Shreveport, Shreveport, LA, United States; ^2^ Department of Cellular Biology and Anatomy, Louisiana State University Health Sciences Center, Shreveport, LA, United States; ^3^ Feist-Weiller Cancer Center, Louisiana State University Health Shreveport, Shreveport, LA, United States

**Keywords:** melanoma, diagnostics, biomarker, liquid biopsy, circulating miRNAs

## Abstract

**Objective:** Melanoma is the most aggressive and deadly form of skin cancer, especially at later stages. There is currently no excellent diagnostic test established for the diagnosis of melanoma; however, circulating microRNAs (miRNAs) have shown some promise. We seek to conduct a systematic review and meta-analysis to establish the clinical utility of circulating miRNAs in diagnosing melanoma.

**Methods:** PubMed, Wiley, and Web of Science were searched for studies that determined miRNA sensitivity and specificity in patients with melanoma. The included studies were assessed in Stata, and the sensitivity, specificity, summary receiver operating characteristic (SROC), positive likelihood ratio, negative likelihood ratio, and the area under the SROC curve (AUC) were calculated.

**Results:** 9 studies with 898 melanoma patients were included in the meta-analysis. The circulating miRNAs showed high diagnostic accuracy with a sensitivity of 0.89 (*p* < 0.001), specificity of 0.85 (*p* < 0.001), diagnostic odds ratio of 45, and an area under the curve of 0.93.

**Conclusion:** Circulating miRNAs have shown a high diagnostic power in detecting melanoma.

## 1 Introduction

From 1990 to 2017, incidence of all diagnosed skin cancers has steadily increased, with approximately 320,000 cases of malignant melanoma diagnosed in 2020, specifically in fair-skinned populations of European descent (Urban et al., 2021; Arnold et al., 2022). In the United States, skin cancer represents 5% of all diagnosed cancers in 2023, with an average annual increase in incidence of 1.2% from 2010 to 2019 (National Cancer Institute, 2023). Cutaneous melanoma, hereafter referred to as melanoma, is a malignancy arising from the preexisting pigment-producing cells located in the skin of individuals known as melanocytes, originating from the neural crest during development (Saginala et al., 2021; Strashilov and Yordanov, 2021).

Several risk factors for melanoma have been explored in literature. The most significant contributor to melanoma formation is ultraviolet (UV) radiation, with 95% of diagnosed cases linked to UV exposure. (Gandini et al., 2005a; Islami et al., 2018). The entire UV spectrum is classified as a carcinogen due to its association with melanoma; hence, limiting UV exposure and using sunscreen is highly recommended as primary prevention (Autier and Doré, 2020; Dzwierzynski, 2021). Additionally, the risk of developing melanoma is notably higher in fair-skinned races when compared to darker skin tones (Lopes et al., 2021). Lastly, 8%–12% of melanoma cases are associated with a positive family history or specific genetic mutations, such as the CDKN2A mutation (Manson et al., 2000; Gandini et al., 2005b; Rossi et al., 2019).

The current diagnostic process for melanoma begins with identification of a suspicious lesion, often incidentally or through self-skin examination. Dermatologist then apply specific criteria and visual inspection techniques with the naked eye or dermoscopy (Kato et al., 2019). Dermoscopy is a technique that uses cross-polarized light and a magnifier placed on skin covered with liquid to see structures in the epidermis and superficial dermis that are not visible to the naked eye, thus allowing for better recognition of the signs of melanoma (Ankad et al., 2020). If the lesion requires additional investigation, an incisional (partial) or excisional (complete) biopsy is performed for histology, with excisional biopsy being the standard of care (Shellenberger et al., 2020).

Visual inspection by dermatologist shows high sensitivity (96.6%) and low specificity (32.3%), resulting in more frequent invasive biopsies with, an average, of 15.6 biopsies to diagnose one case of melanoma (Nelson et al., 2019; MacLellan et al., 2021). Additionally, an excisional biopsy can leave a large scar, require surgical fixation with a skin flap or can be difficult to perform in challenging areas, where an incisional biopsy is performed instead (Restrepo et al., 2019; Naik, 2021). Additionally, dermoscopy results in a limited diagnostic accuracy due to low resolution and lack of optical sectioning capabilities (Wang and Evans, 2016). Finally, a crucial prognostic indicator for treatment in melanoma is the early detection and treatment of the cancer, yet there is no approved screening recommended by the US Preventative Services Task Force (Mohammadpour et al., 2019; O'Neill and Scoggins, 2019; Arnold et al., 2022). However, in some populations, diagnosis is delayed by as long as 9 months, potentially due to inadequate education or irregular skin exams by physicians (Naik, 2021). Overall, our current diagnostic schema for early detection and diagnosis of melanoma is nonspecific and involves invasive procedures.

As advancements in precision medicine become clinically more apparent, medicine shifts towards a more individualized approach such as utilizing liquid biopsy to characterize the molecular features of a patient’s tumor to monitor therapeutic outcomes, recurrence, predict prognosis, and diagnose various cancers (El-Deiry et al., 2019; Ho et al., 2020; Tsimberidou et al., 2020; Alix-Panabières and Pantel, 2021). Liquid biopsy remains less invasive than excisional biopsy or other test requiring intravenous contrast by requiring only access to a peripheral vein for a blood draw (Perakis and Speicher, 2017; Lianidou and Pantel, 2019; Underwood et al., 2020).

Liquid biopsy utilizes various genetic material from cells that circulate in the blood, specifically the microRNAs (miRNAs), a small non-coding genetic material responsible for epigenetic regulation and maintains a pivotal role in oncogenesis (Tengda et al., 2018; Buscail et al., 2019; Sabato et al., 2022). The expression of miRNAs is altered due to mutations in the genetic code caused by the tumor, leading to the overexpression of oncogenic miRNAs or the under-expression of tumor-suppressing miRNAs (Gajos-Michniewicz and Czyz, 2019). The altered expression of miRNA levels have been established as a prognostic indicator correlating with survival time or relapse in patients (Lu et al., 2019; Dika et al., 2020). Furthermore, miRNA levels impact medical management because their levels can be used to gauge treatment effectiveness (Svedman et al., 2018; Kamińska et al., 2021). However, the clinical efficacy of using altered expression of miRNA in the diagnosis of melanoma is not established.

This systematic review and meta-analysis aim to collect published data on upregulated miRNAs in melanoma patients. We seek to establish clinical viability of circulating miRNAs as a less invasive alternative for diagnosing melanoma, reducing reliance on excisional biopsies.

## 2 Materials and methods

### 2.1 Search strategy

A comprehensive search across Web of Science, PubMed, and Wiley was conducted utilizing broad terms to encompass various articles without premature exclusion. All publications were searched for in English, and an additional reference list was screened to incorporate additional studies. The search strings consisted of “melanoma” and “microRNA” and (“diagnosis” OR “early detection” or “sensitivity and specificity”) and filtered to include articles within the last 13 years only.

### 2.2 Eligibility criteria

Our study selection criteria included studies reporting upregulated miRNA in the blood of melanoma patients, detailing with sensitivity and specificity for diagnosing melanoma, and specifying the total number of participants. Conference abstracts, unpublished articles, review letters to the editor, animal studies, and studies with subjects fewer than 10 were not within the scope of our analysis and consequently excluded.

### 2.3 Data extraction and quality assessment

The studies were full-text reviewed for the following information: 1) study characteristics (sample size, year, and author); 2) miRNA features (type of miRNA studied, dysregulated expression, detection methods, and sample types); 3) analysis data (sensitivity and specificity or TP, FN, TN, FP data).

The quality of each study was assessed using the Quality Assessment of Diagnostic Accuracy Studies (QUADAS-2) criteria (Whiting et al., 2011). Studies that received a high-risk evaluation for a particular signaling question in any domain were considered to have a high risk of bias within that domain. The four key domains were patient selection, index test, reference standard, and flow and timing. While all four domains were assessed for risk of bias, the first three were additionally evaluated for outside applicability.

### 2.4 Statistical analysis

The meta-analysis is conducted using STATA/BE (v.18.0) and RevMan (v. 5.4) statistical software. A random-effects model is used to calculate sensitivity, specificity, positive likelihood ratio (PLR), negative likelihood ratio (NLR), and diagnostic odds ratio (DOR) with pooled 95% confidence intervals. The diagnostic accuracy of circulating miRNAs is assessed using forest plots and summary receiver operating characteristics (SROC) and the area under the curve (AUC). The Cochrane’s Q test and I^2^ statistics are used to assess the presence of statistical heterogeneity between studies. A Deek’s funnel asymmetry plot is used to assess publication bias.

## 3 Results

### 3.1 Study selection

**FIGURE 1:**
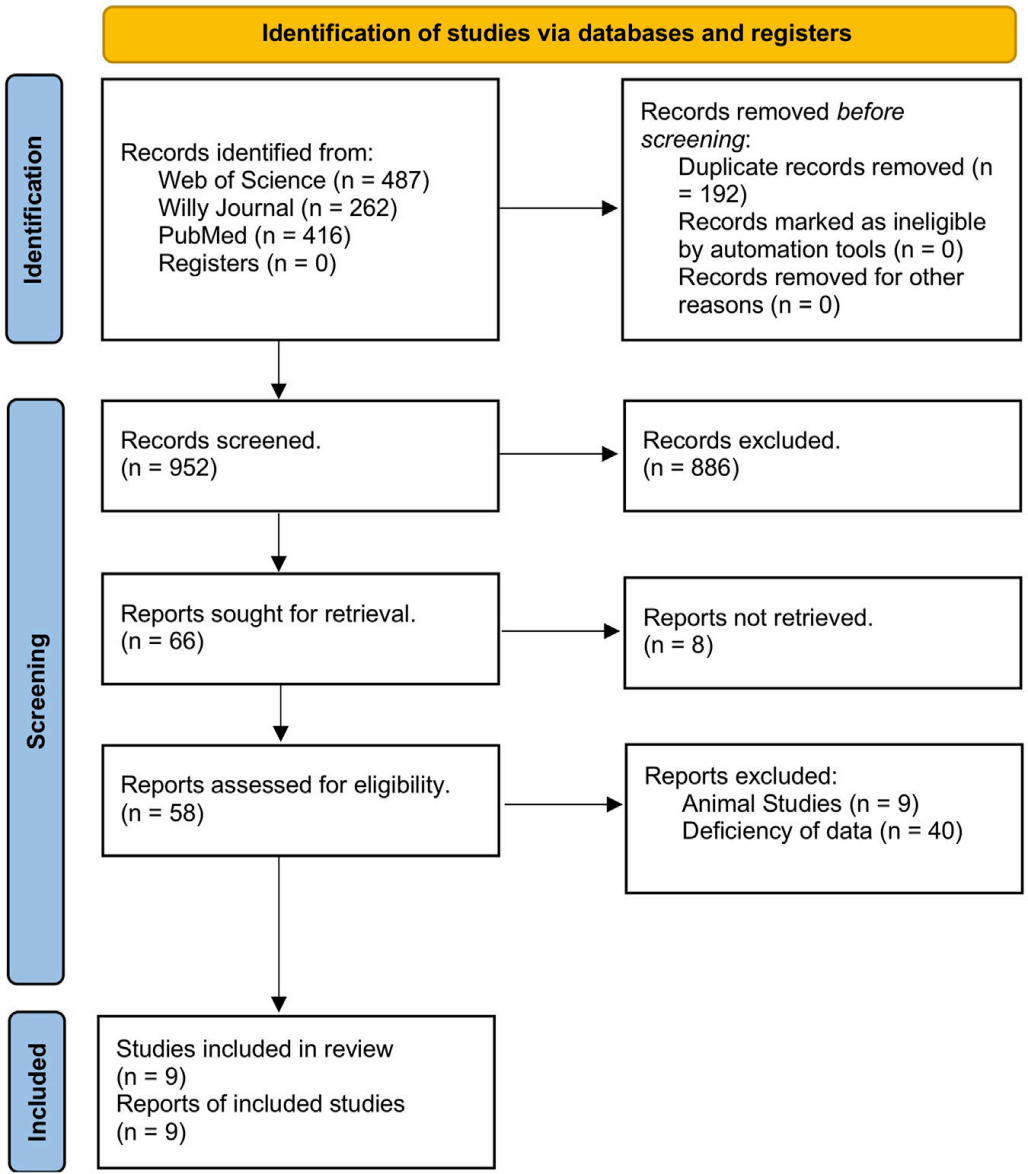
PRISMA flowsheet on the study selection process.

An initial literature search yielded 1,144 studies across the three databases used: Web of Science, Wiley, and PubMed. Among those, 192 articles were removed due to being duplicates, leaving 952 studies to be screened. After the title review, 952 studies were deemed irrelevant and excluded. Of the 66 articles, eight were inaccessible for retrieval. Finally, nine studies (Leidinger et al., 2010; Stark et al., 2015; Armand-Labit et al., 2016; Bai et al., 2017; Fogli et al., 2017; Tengda et al., 2018; Van Laar et al., 2018; Mo et al., 2019; Van Laar et al., 2023) demonstrating the upregulation of miRNAs were selected for inclusion in the meta-analysis. The screening process is outlined in Figure 1.

### 3.2 Study characteristics

The nine eligible studies included 898 patients with melanoma and 556 healthy controls. Four studies collected plasma samples, four used serum samples, and one used blood samples to collect patient specimens. All studies used quantitative reverse transcription-polymerase chain reaction (qRT-PCR) to detect miRNA levels. Six of the studies used a panel of multiple miRNAs throughout their investigations. All study characteristics are outlined in Table 1.

**TABLE 1:** Summary of characteristics from studies included in the meta-analysis.

Author (Year)		Sample size							
	Year	Patients	Control	miRNA studied	TP	FP	FN	TN	Sample
Leidinger et al. (2010)	2010	35	20	16 miRNAs panel^a^	34	1	1	19	Blood
Stark et al. (2015)	2015	255	130	17 miRNAs panel^b^	237	23	18	107	Serum
Armand-Labit et al. (2016)	2016	31	43	miR-1246 + miR-185	28	5	3	38	Plasma
Bai et al. (2017)	2017	85	30	miR-10b	65	4	20	26	Serum
Fogli et al. (2017)	2017	30	32	miR-149-3p	28	4	2	28	Plasma
Fogli et al. (2017)	2017	30	32	miR-150-5p	29	10	1	22	Plasma
Fogli et al. (2017)	2017	30	32	miR-193a-3p	23	12	7	20	Plasma
Fogli et al. (2017)	2017	30	32	miR-15b-5p	27	7	3	25	Plasma
Fogli et al. (2017)	2017	30	32	miR-524-5p	27	10	3	22	Plasma
Tengda et al. (2018)	2018	25	25	miR-532-5p + miR-106b	23	3	2	22	Serum
Van Laar et al. (2018)	2018	35	22	38 miRNAs panel^c^	25	3	10	19	Plasma
Mo et al. (2019)	2019	60	40	miR-21	47	7	13	33	Serum
Van Laar et al. (2023)	2023	372	210	38 miRNAs panel^c^	346	4	26	206	Plasma

TP, true positive; FP, false positive; FN, false negative; TN, true negative. ^a^16 miRNAs panel comprises miR-186, let-7d, miR-18a, miR-145, miR-99a, miR-664, miR-501-5p, miR-378, miR-29c, miR-1280, miR-365, miR-1249, miR-328, miR-422a, miR-30d, and miR-17. ^b^17 miRNAs panel comprises miR-211-5p, miR-514a-3p, miR-509-3p, miR-204-5p, miR-509-5p, miR-513b, miR-145-5p, miR-146a-5p, miR-508-3p, miR-506-3p, miR-513c-5p, miR-4731-5p, miR-508-5p, miR-363-3p, miR-4487, miR-4706, and miR-16. ^c^38 miRNAs panel comprises miR-424-5p, miR-548l, miR-34a-5p, miR-497-5p, miR-299-3p, miR-205-5p, miR-1269a, miR-624-3p, miR-138-5p, miR-1-5p, miR-152-3p, miR-1910-5p, miR-181b-5p, miR-3928-3p, miR-3131, miR-301a-3p, miR-1973, miR-520d-3p, miR-548a-5p, miR-548ad-3p, miR-454-3p, miR-4532, miR-1537-3p, miR-553, miR-764, miR-1302, miR-1258, miR-522-3p, miR-1264, miR-1306-5p, miR-219a-2-3p, miR-431-5p, miR-450a-5p, miR-2682-5p, miR-337-5p, miR-27a-3p, miR-4787-3p, and miR-154-5p.

### 3.3 Quality assessment

**FIGURE 2:**
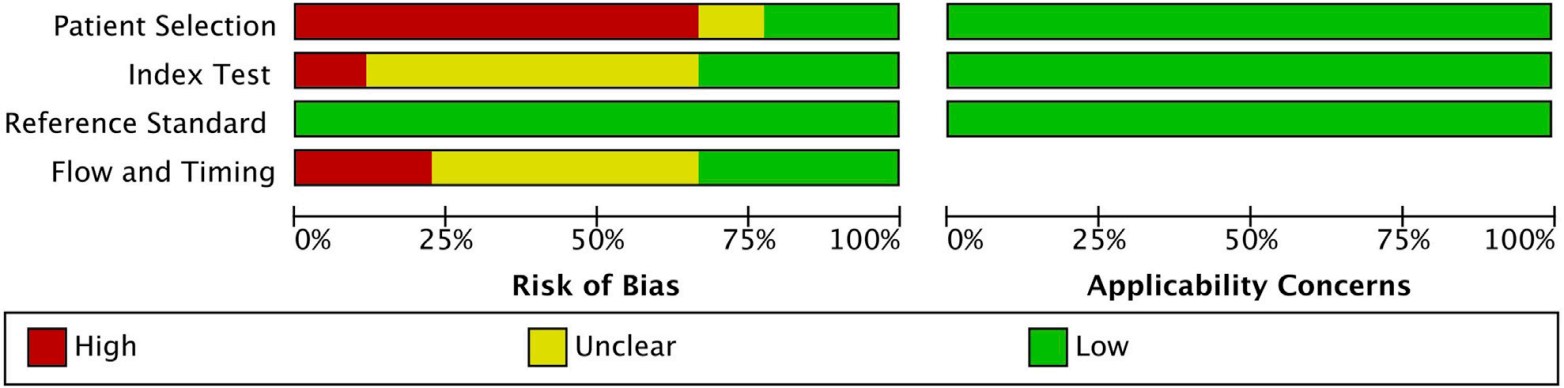
QUADAS-2 quality assessment of the 9 included articles.

A detailed assessment of the quality of the 9 included studies is provided in Figure 2. All studies followed a case-control design, and a few studies employed randomized selection, resulting in a high risk of patient selection bias. The included studies exhibited low risk of bias for the index test and reference bias, while the risk for flow and timing was unclear. Therefore, overall study quality is deemed acceptable for this meta-analysis.

### 3.4 Meta-analysis

**FIGURE 3:**
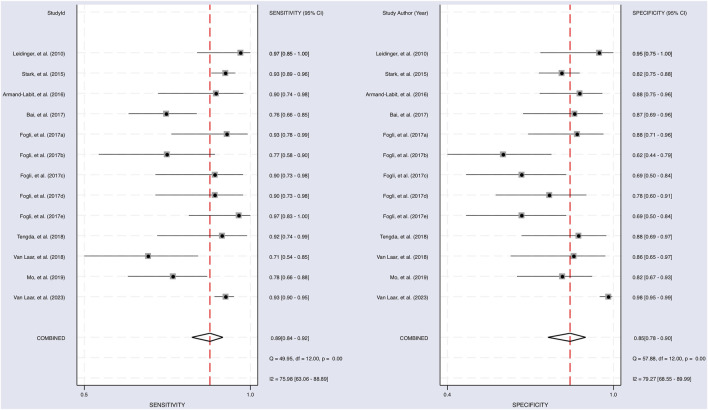
Sensitivity and specificity of upregulated miRNA in the diagnosis. The sensitivity of each study with a pooled sensitivity on the left and the specificity of each study with a pooled specificity on the right.

**FIGURE 4:**
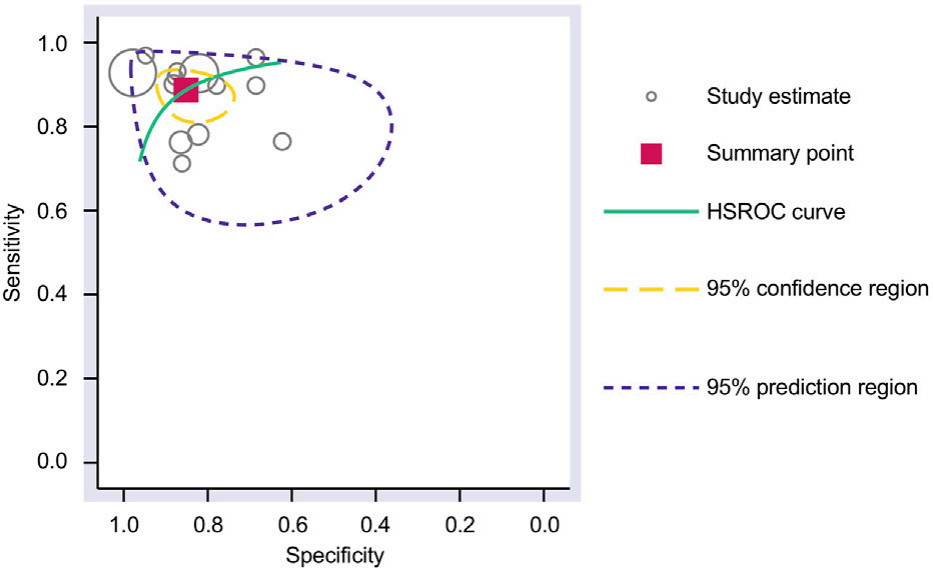
SROC curve analysis. The AUC for the SROC was 0.93 (95% CI 0.91–0.95) for using upregulated miRNA blood tests in the diagnosis of melanoma. AUC, area under the curve; SROC, summary receiver operating characteristic.

**FIGURE 5:**
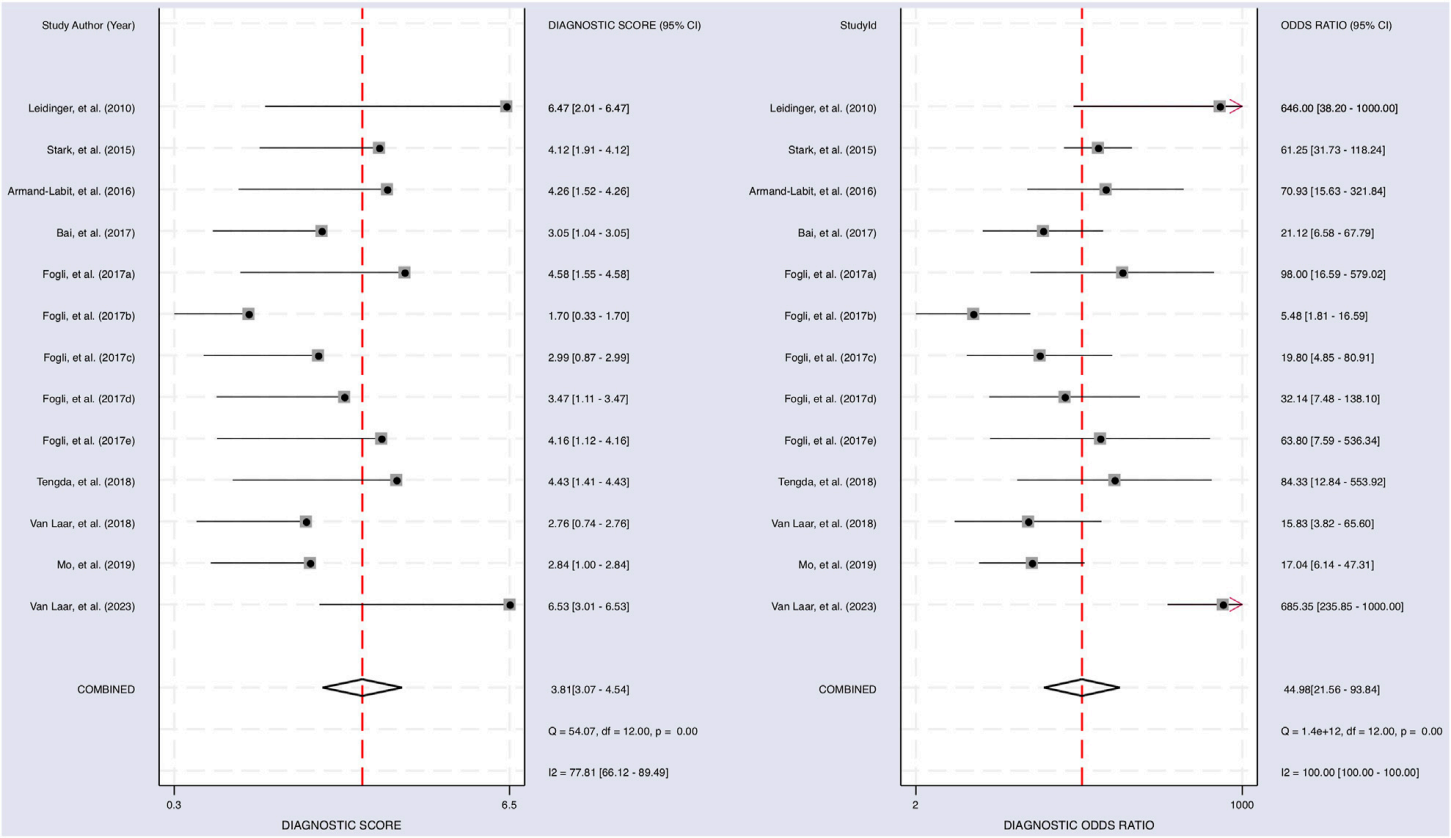
The diagnostic performance of miRNAs on predicting melanoma. Forest plot of individual and pooled diagnostic scores (left) and diagnostic odds ratio (right) of the included 9 articles.

**FIGURE 6:**
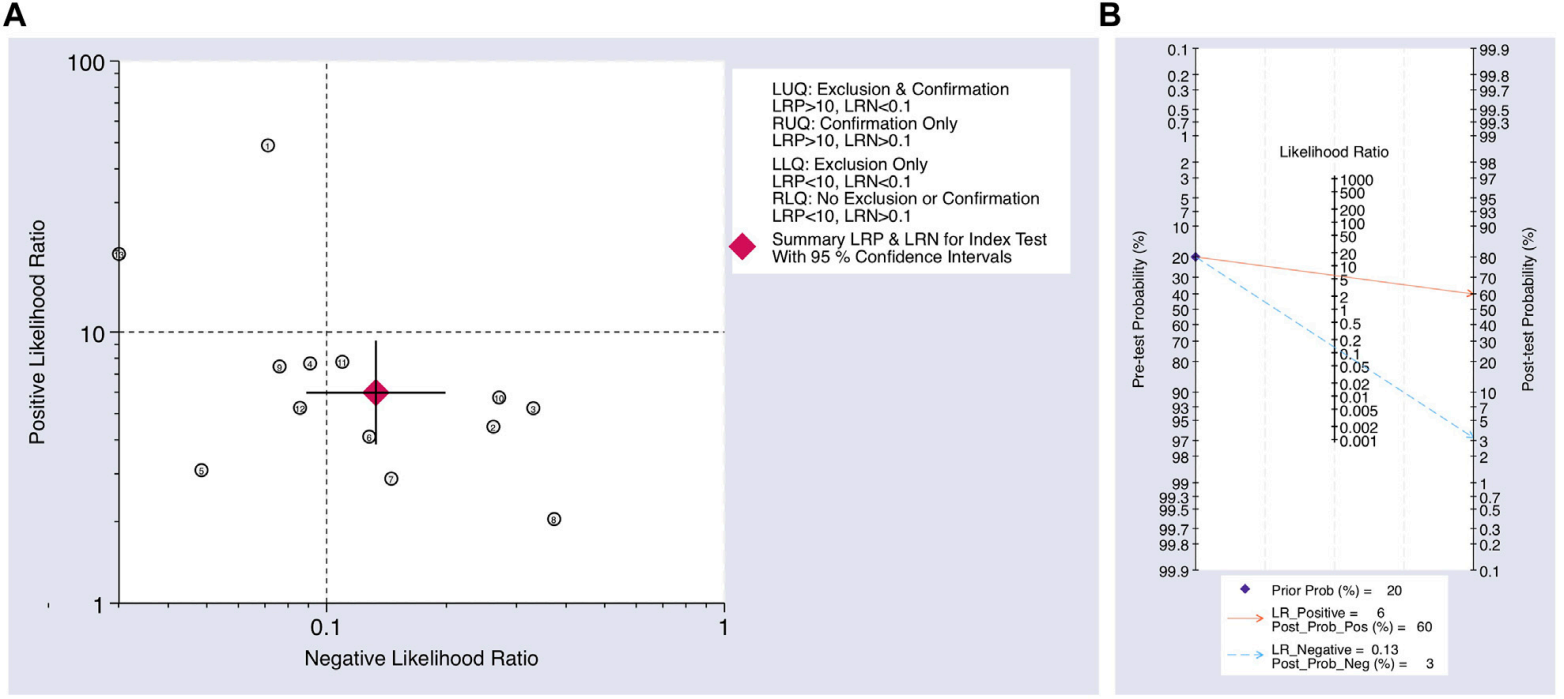
**(A)** Scatter plot using the NLR and PLR. **(B)** Fagan’s nomogram for post-test probability using the NLR and PLR for using upregulated miRNAs as a biomarker in the blood for the diagnosis of melanoma.

The Cochrane’s Q test and I^2^ test showed that the I^2^ values were 75.98 [95% CI 63.06–88.89] and 79.27 [95% CI: 68.55–89.00]. Hence, a random-effects model was used for the statistical analysis, as heterogeneity was detected within the studies. A Forest Plot illustrates the studies’ individual and overall pooled sensitivity and specificity, as shown in Figure 3. The pooled sensitivity of 0.89 (95% CI: 0.84–0.92, *p* < 0.001) and specificity of 0.85 (95% CI: 0.78–0.90, *p* < 0.001). The AUC for the SROC curve analysis shown is 0.93 (95% CI: 0.91–0.95), indicating a high accuracy (Figure 4). The pooled DOR was 44.98 (95% CI: 21.56–93.84), shown in Figure 5. The PLR and NLR were 5.98 (95% CI: 3.85–9.30) and 0.13 (95% CI: 0.09–0.20), respectively. The PLR and NLR scattergram with the effects on post-test probability are shown in Figure 6.

### 3.5 Publication bias

**FIGURE 7:**
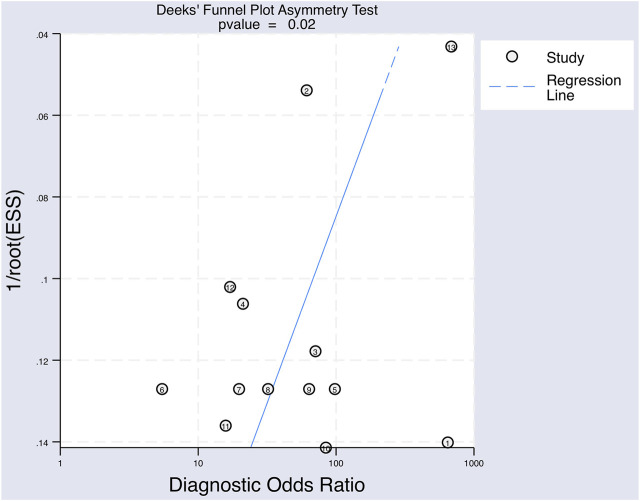
The Deeks’ funnel plot asymmetry test for publication bias of the included studies testing for upregulated miRNAs for the diagnosis of melanoma.

A Deek’s Funnel Plot Asymmetry Test was used to evaluate publication bias within the eligible studies and showed a *p*-value of 0.02 (Figure 7), which indicates significant publication bias.

## 4 Discussion

Melanoma is the most aggressive and lethal form of skin cancer, particularly in advanced stages. Melanoma can appear on skin not normally exposed to the sun, and if untreated, it swiftly escalates into a life-threatening condition within a mere 6 weeks.

The most misdiagnosed lesion is amelanotic melanoma. An aggressive subtype of melanoma that does not produce the pigment melanin, it can be easily misidentified as harmless scars or mole. Patients may miss the opportunity to be fully cured because amelanotic melanomas often spread faster than the more easy-to-recognize melanomas. Therefore, the combination of routine examination of the skin using dermoscopy, coupled with liquid biopsy, provides a realistic opportunity for the detection of malignant melanomas including amelanotic forms.

Early detection of melanoma can improve prognosis for patients significantly. Key to early detection is the identification of biomarkers, and this is facilitated *via* obtaining liquid biopsy samples that contain tumor-derived materials such as miRNA (Nonaka and Wong, 2023). miRNAs play an essential role in biological processes by regulating gene expression at the post-transcription level. miRNAs bind to messenger RNA (mRNA) in the cytoplasm, resulting in mRNA degradation or temporary inhibition of translation. miRNAs contribute to nearly all aspects of cancer biology, including proliferation, differentiation, angiogenesis, and metastasis. Their expression is frequently dysregulated in cancer, creating a distinct expression profile. Each cancer possesses a specific miRNA expression profile, either overexpressed miRNAs targeting tumor-suppressor genes or downregulated miRNAs targeting oncogenes (Sethi et al., 2014). This miRNA expression profile could potentially be used to establish biomarkers capable of identifying specific cancer types.

Circulating miRNAs are being considered as promising biomarkers for many human diseases since they fulfill several criteria for being preferable biomarkers. These circulating miRNAs are enriched in extracellular vesicles, and they are stable and protected from endogenous RNase activity (Valadi et al., 2007). Additionally, miRNAs demonstrate high specificity for tissue or cell types (Jung et al., 2012; Wang et al., 2012; Wu et al., 2012). Distinct miRNA profiles can be identified for different cancer types, which could then serve as phenotypic signatures (Ahmad et al., 2014).

This study aimed to evaluate the clinical utility of using liquid biopsy of upregulated circulating miRNAs to diagnose melanoma. This study has shown that using blood levels is a highly sensitive and specific test with pooled values of 0.89 and 0.85, respectively, highlighting the strengths of using miRNAs as a clinical test in diagnosing melanoma. The NLR or PLR, 0.13 and 5.98, respectively, have moderate effects on the shift from pre-test probability to post-test probability (Jaeschke et al., 1994). The AUC of the SROC curve was 0.93, indicating that using upregulated miRNA levels in the blood for diagnosing melanoma is an excellent test (Šimundić, 2009). Additionally, this study adds to a gap in the literature on using miRNA to diagnose melanoma and can lead to advances in precision oncology, proving that a less invasive liquid biopsy for upregulated miRNA could become the new gold standard for melanoma diagnosis instead of an invasive excisional biopsy.

Although our meta-analysis demonstrated the potential use of upregulated miRNAs in diagnosing melanoma, certain limitations persist in their application. First, significant heterogeneity was observed among the included studies. Second, specific patient characteristics that could affect circulating miRNA levels, such as other comorbidities or ongoing treatments of patients during the blood draw, were not consistency recorded across studies. Future studies should prioritize standardizing the method of miRNA collection, determine the specific strands for evaluating patients suspected of melanoma, and consider alternative participant selection methods, moving away from the case-control format to increase validity in larger, diverse populations.

A growing body of evidence implicates the clinical utility of circulating biomarkers extracted from multiple body fluids (e.g., blood, saliva, urine) for cancer patients, focusing on patient stratification and monitoring disease status (Nonaka and Wong, 2018). The use of circulating biomarkers for cancer detection, diagnosis, and disease monitoring is an exciting prospect (Nonaka and Wong, 2022). Indeed, our study illustrates a high diagnostic accuracy of circulating miRNAs in diagnosing melanoma. These findings further reinforce the notion that precision medicine in oncology is advancing and that less invasive testing can achieve similar or better results compared to the established paradigm for diagnosing melanoma.

## Data Availability

The original contributions presented in the study are included in the article/Supplementary material, further inquiries can be directed to the corresponding author.
